# High-Identical Numerical Aperture, Multifocal Microlens Array through Single-Step Multi-Sized Hole Patterning Photolithography

**DOI:** 10.3390/mi11121068

**Published:** 2020-11-30

**Authors:** Joong Hoon Lee, Sehui Chang, Min Seok Kim, Yeong Jae Kim, Hyun Myung Kim, Young Min Song

**Affiliations:** School of Electrical Engineering and Computer Science, Gwangju Institute of Science and Technology (GIST), 123 Cheomdangwagi-ro, Buk-gu, Gwangju 61005, Korea; dlwndgns2@gist.ac.kr (J.H.L.); shchangj@gm.gist.ac.kr (S.C.); seok9643@gist.ac.kr (M.S.K.); kimyeongjae@gm.gist.ac.kr (Y.J.K.); gusaud31@gist.ac.kr (H.M.K.)

**Keywords:** optical MEMS, microlens array, multiple focal lengths, three-dimensional imaging

## Abstract

Imaging applications based on microlens arrays (MLAs) have a great potential for the depth sensor, wide field-of-view camera and the reconstructed hologram. However, the narrow depth-of-field remains the challenge for accurate, reliable depth estimation. Multifocal microlens array (Mf-MLAs) is perceived as a major breakthrough, but existing fabrication methods are still hindered by the expensive, low-throughput, and dissimilar numerical aperture (NA) of individual lenses due to the multiple steps in the photolithography process. This paper reports the fabrication method of high NA, Mf-MLAs for the extended depth-of-field using single-step photolithography assisted by chemical wet etching. The various lens parameters of Mf-MLAs are manipulated by the multi-sized hole photomask and the wet etch time. Theoretical and experimental results show that the Mf-MLAs have three types of lens with different focal lengths, while maintaining the uniform and high NA irrespective of the lens type. Additionally, we demonstrate the multi-focal plane image acquisition via Mf-MLAs integrated into a microscope.

## 1. Introduction

Over the last few decades, microlens arrays (MLAs) have become an indispensable optical component, owing to their wide field-of-view (FOV) and simple configurations, and are applied in a wide range of fields, such as bio-inspired compound eye systems [1,2,3,4], computational holograms [5,6], and surveillance cameras [7,8]. In particular, the multiple parallax images from the MLAs have attracted the attention of many research groups working on the application of 3D imaging, and particularly 3D medical imaging systems [9,10], and light field imaging [11,12,13]. However, to actively apply the MLAs in 3D imaging systems, the depth-of-field (DoF) of the conventional MLAs should be sufficiently enlarged for achieving an accurate depth estimation. The DoF can be widened by lowering the lens’ sag-height [14], which has a low numerical aperture (NA), but it is noted that an accurate depth estimation and a high optical resolution are mutually incompatible from the wide DoF and low NA. As an alternative approach, MLAs with multiple foci can resolve this limitation, achieving not only the accurate depth estimation, but also the high optical resolution [15,16].

In the fabrication of wafer-level MLAs, the MEMS-based precision machining method and the resistance to thermal reflow are commonly employed as main fabrication techniques [17,18]. In addition, the combined and tweaked fabrication methods allow for the design of MLAs with various functions, such as aspheric MLAs [19], high NA [20], or and antireflection [21,22]. For multifocal MLAs, several approaches were newly demonstrated, such as a guided resistance to thermal reflow and multi-stacked microposts [23,24], which cannot be broadly used because of the multiple photolithography step, sophisticated align process, high cost, non-uniform NA among lenses, and low yield rate. Moreover, for utilizing the multifocal MLAs in 3D depth estimation systems (e.g., light field camera), uniform NA among lenses with different foci is required to reduce the computational cost [11]. Hence, the above-mentioned limitations demand a novel fabrication technique for high and identical NA and cost-effective, multifocal MLAs.

In this study, we propose a novel wafer-level fabrication method for multifocal MLAs (Mf-MLAs) with steady and high NA through one-step photolithography. The fabricated Mf-MLAs have a high NA, of ~0.43, which attains not only a high optical resolution, but also an accurate depth estimation through the extended DoF. Mf-MLAs’ mold can be readily fabricated at the wafer level by the isotropic chemical wet etching of multi-sized holes created by single-step photolithography. Individual lens’ design parameters such as radius of curvature and lens diameter are satisfied by varying the etch time during the chemical wet etching process.

## 2. Materials and Methods

### Design and Fabrication of Mf-MLA

**Figure 1:**
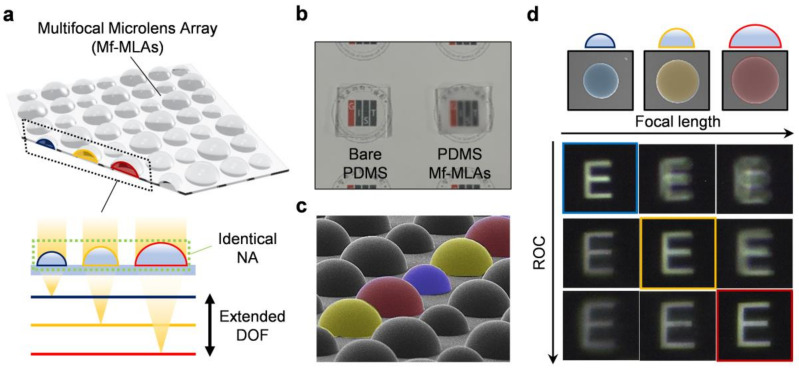
(**a**) Concept schematic of Multifocal Microlens Arrays (Mf-MLAs) with three different radii of curvature (RoC) for an extended depth of field. (**b**) Photographs of bare poly-dimethylsiloxane (PDMS) and a replicated PDMS Mf-MLAs sample. (**c**) 40-degree tilted view SEM image. (**d**) Microscope images of letter “E” placed on object distances corresponding to each lens’ focal length.

Conventional MLAs with a single focal length inevitably have a narrow DoF, which leads to the restriction of the accurate depth estimation from the parallax images of individual lenses. On the contrary, Mf-MLA is composed of several types of lenses with different focal lengths, so that it is possible to collect the parallax images from two or more object planes in different distances. Thus, the Mf-MLA provides an extended DoF, as shown in Figure 1a, enabling us to get accurate depth information on the target object. For the lens’ material, we were particularly concerned with two features: flexibility and compatibility. To this end, poly-dimethylsiloxane (PDMS, Sylgard 184, Dow Corning Corporation, Midland, MI, USA) was selected as a representative material for our Mf-MLA. Figure 1b shows the comparison between bare PDMS and PDMS Mf-MLAs, and they have a good compatibility with other optoelectronic elements, such as an image sensor array and glass or plastic optics, easily attachable by van der waals force [25]. The “GIST” logo is clearly imaged under the bare PDMS (Figure 1b; left). In contrast, the fabricated Mf-MLAs shows a blurred image owing to the microscale structure at the surface, but maintains the color information beneath the Mf-MLAs (Figure 1b; right).

The shaping of the lens’ surface is conducted mainly by the wet etching process. The Mf-MLA mold is isotropically etched forming the nearly hemispherical-shaped lens surface regardless of its size, which allows for high-NA Mf-MLAs, providing not only an accurate depth estimation but also a high optical resolution. Focal lengths in Mf-MLA are modulated by the different hole-size patterns in the photolithography step. Note that the different false-color (red, yellow, blue) indicates the three types of lenses in Mf-MLA formed by a different hole-size patterning and the same etch time (Figure 1c). Figure 1d shows images of letter “E” as an object through the engineered Mf-MLA, photographed by the microscope system. According to each focal length of the lenses, the object is clearly focused from each object plane.

**Figure 2:**
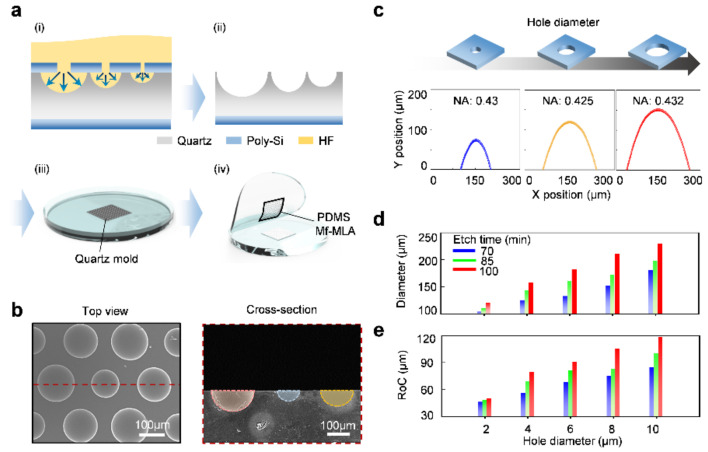
(**a**) Process schemes for Mf-MLA fabrication with three different radii of curvature (RoC). Diverse lens diameters of Mf-MLA are controlled by the via-hole size. (**b**) SEM image of the fabricated quartz master mold with different viewing angle, Top view; (left) and cross-sectional view (right). (**c**) The measured morphology profiles of replicated PDMS Mf-MLA with different via-hole diameters. (**d**) The diameter changes of each microlens according to the wet etch time. (**e**) The RoC change of each microlens according to the wet etch time.

The Mf-MLA fabrication process can be conducted by two primary steps: multi-sized hole patterning photolithography and chemical wet etching. First, the Mf-MLAs’ mold is patterned with multi-sized holes by single-step photolithography of positive photoresist (PR, AZ 5214, AZ Electronic Materials, Co., Ltd, Luxembourg), and then the wet etching process is carried out, consecutively (Figure 2a). The entire fabrication procedure of Mf-MLA is as follows: (i) 700 nm thick Poly-Si is deposited on a quartz substrate in order to prevent the penetration of hydrofluoric acid (HF) in the next wet etching process. Hole-patterning was performed by using a multi-sized hole mask on one side of the Poly-Si through photolithography. Note that we set 2 μm as the minimum hole size, due to the resolution (refer as the minimum critical dimension) of the mask aligner (M100, Prowin, Incheon, Korea) using our demonstration. (ii) Dry-etching via an inductively coupled plasma reactive ion etch (ICP-RIE) is conducted on the patterned Poly-Si sample, according to the etching conditions as follows: SF6 flow/working pressure/RF power/ICP power/etch time = 50 sccm/4 mTorr/50 W/100 W/2 min. (iii) By dipping the sample in an HF solution, the quartz substrate is isotropically etched based upon the guide of the patterned hole of the Poly-Si. Note that the maximum pitch of adjacent microlenses is predefined by the lithography mask of the previous step. After the Poly-Si is eliminated, by using a potassium hydroxide (KOH) solution, a concave Mf-MLA quartz mold is created with the multiple curvatures and sag heights. (iv) A PDMS replica molding is conducted on the concave Mf-MLA quartz mold. To demold the cured PDMS Mf-MLA, a fluorocarbon-based release agent is used as an anti-adhesive spray (DAIFREE GA-7550, Daikin, Japan). The spraying distance from the mold is ~30 cm, and the agent is spread evenly for 5 s. Next, PDMS is poured and cured on the concave Mf-MLA mold at room temperature for ~4 d, preventing the thermal deformation of the PDMS. After the polymer solidification, PDMS Mf-MLA is gently peeled off from the mold.

In Figure 2b, the scanning electron microscope (SEM, Hitachi S-4700, Hitachi, Tokyo, Japan) images show the geometrical information of the fabricated master quartz mold for Mf-MLA (Figure 2a; (ii)). The cross sectional view (Figure 2b; right) from the dashed deep-red line in the top view (Figure 2b; left) clearly shows that each lens is formed with different lens diameters. Likewise, the pitch and geometric shape (i.e., 200 μm and hemispheric form, respectively) of adjacent lenses satisfies the designed value. For the quantitative analysis, the fabricated Mf-MLA was scrutinized by using a confocal laser scanning microscope (CLSM). Figure 2c represents the surface morphology variation according to the change in the via-hole diameter at the same wet etch time. Moreover, the results show a fine surface roughness (arithmetical mean deviation, R_a_) below 0.045 μm (Table 1). The wet etch time is 85 min and each diameter of the hole is 2, 6 and 10 μm, respectively. As the via-hole diameter increased, microlens was formed with enlarged radius and sag height. In addition, the diameter and radius of curvature (RoC) of individual microlenses were also measured along with the via-hole diameter variation (Figure 2d,e), indicating that lens parameters such as RoC and diameter are easily manipulated through via-hole diameter and the wet etch time.

## 3. Results and Discussion

### 3.1. Characterization of Mf-MLA

**Figure 3:**
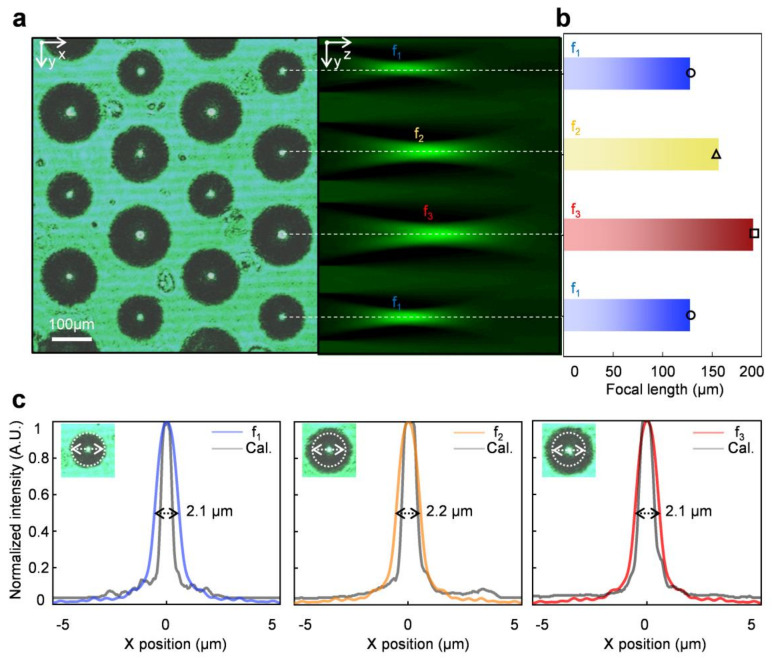
(**a**) Three experimentally measured focal lengths of Mf-MLAs through the stacked optical microscope images (top view; left, cross-sectional view; right). The pitch between microlenses is 200 μm. (**b**) Graph of measured (bars) and calculated (symbols) focal lengths at each microlens with three different focal lengths. (**c**) Intensity profiles along the X-axis of the foci depending on the lens type of the fabricated Mf-MLA.

The multifocal features of the fabricated Mf-MLA were measured by using a microscope system and an image stacking method with a commercial software (MATLAB, Mathworks, Natick, MA, USA), under a collimated laser beam at 532 nm. The focusing ability of the fabricated Mf-MLA is characterized on the motorized stage microscope by sequentially imaging the estimated range of focal plane at an interval of 1 μm along the optical axis (z-plane) of the Mf-MLA. The top view (x-y plane) image indicates the fine spatial uniformity of beam spots at the foci of the Mf-MLA. The obtained 3D optical sectioning of the top-view and stacked optical images is the Mf-MLA for 200 μm in pitch under the 85-min etch time. The optical sections in the y-z plane also show that the focal length (f) of Mf-MLAs decreases as the diameter increases (Figure 3a). The measured focal lengths of Mf-MLA were 128, 157, and 192 μm fabricated under the 85-min etch time from hole pattern with diameters of 2, 6, and 10 μm, respectively. The experimental results are well matched with the calculated focal lengths of 130, 155, and 194 μm, based on the results in Figure 2e and the approximated lens maker formula, f = RoC/(n-1), where n is the refractive index (Figure 3b). Then, the point spread function (PSF) was elicited from the beam spots of each type of the microlens. The PSFs clearly show that each lens has a similar beam-spot diameter of ~2.1 μm (full width at 1/e2 maximum) regardless of the focal length increase from 128 to 192 μm. This can be explained by the identical NA of the Mf-MLA, which is enabled by the isotropic wet etching processes. The measured PSFs are also well matched to the calculated PSFs from the ray-tracing simulation based on the Monte Carlo method (Figure 3c–e).

### 3.2. Multifocal Arrayed Images

**Figure 4:**
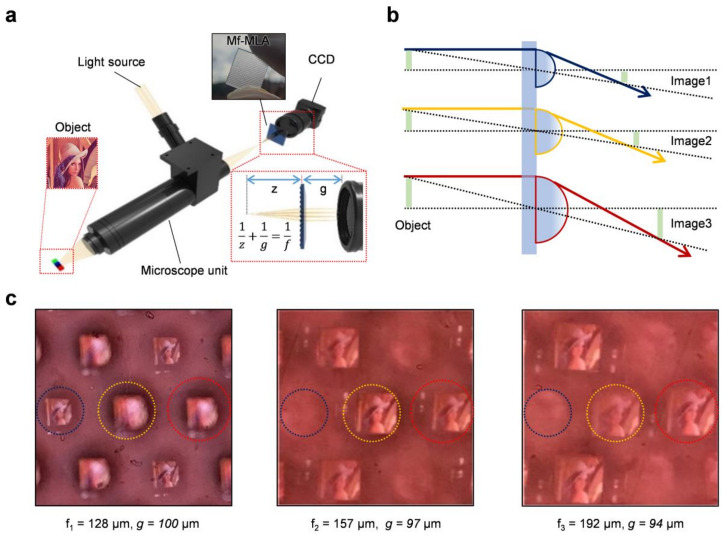
(**a**) Schematic illustration of the optical setup for capturing micro-images corresponding to the focal lengths of each lens type of Mf-MLA. An object, main optics, Mf-MLAs, and a CCD image sensor were precisely aligned along the optical axis. (**b**) Illustrations of the magnification of each micro-image depending on the focal length of each lens of the Mf-MLA. (**c**) Photographs of images through the fabricated Mf-MLA at the optimized positions of each lens type between the Mf-MLAs and the image sensor.

For the practical analysis, we constructed a light field imaging system to capture multifocal images formed by fabricated Mf-MLAs. Figure 4a depicts the schematic of the light field microscopy system using Mf-MLA, which is adopted in this study. The angular information can be extracted by the Mf-MLA along the x-axis, which has the intermediate image formed by the main optics as an object. A Lena image (1 mm × 1 mm) was used as an object for the light field imaging test at three different focal points. The imaging system operates as a relay optics between main optics and an individual micro-image, which operate as a virtual camera. The object was precisely placed to align with a 5.1-megapixel complementary metal-oxide-semiconductor image sensor array (CMOS ISA) (SONY IMX 264, SONY Corp., Tokyo, Japan, pixel size = 3.45 μm). To maximize the range of the extended DoF, we adopted the formula for the DoF of the Mf-MLAs and its maximizing condition as the left and right boundary of the three types of lenses. The equation is as follows [26]:

(1) $$a_{0}^{-}={\left[\frac{1}{f}-\frac{1}{g}\left(1-\frac{p}{d}\right)\right]}^{-1}$$

(2) $$a_{0}^{+}={\left[\frac{1}{f}-\frac{1}{g}\left(1+\frac{p}{d}\right)\right]}^{-1}$$

where $a_{0}^{-}$ and $a_{0}^{+}$ are the left and right boundaries of the DoF, g is the distance between the Mf-MLA and the CMOS ISA, d is the diameter of the microlens, and p is the pixel size, respectively.

Then, the DoF is

(3) $$a_{0}=\left|a_{0}^{+}-a_{0}^{-}\right|$$

Thus, the optimized condition for the maximum extended DoF is as follows:

(4) $$a_{0}^{+}\left(f_{1}\right)=a_{0}^{-}\left(f_{2}\right)$$

When the g is the variable, the optimized positions of the Mf-MLAs (*f*_1_ = 128 μm, *f*_2_ = 157 μm, *f*_3_ = 192 μm at *d*_1_ = 110 μm, *d*_2_ = 136 μm, *d*_3_ = 165 μm) are 100, 97, and 94 μm respectively. Figure 4c shows the obtained images at the optimized g of each type of microlens by using a xyz translation stage. The DoF ranges are −783.8 to −316.6 μm, −316.3 to −211 μm, and −210.9 to −164.7 μm for the Galilean light field mode [27]. The results show that the Mf-MLAs reached the ~6 times extended whole DoF range from 105.3 μm to 619.1 μm, implying the enlarged depth estimation range in the light-field system.

## 4. Conclusions

In summary, we present a simple, but clever fabrication of multifocal microlens arrays (Mf-MLAs) with high numerical aperture and extended DoF. The systematic fabrication of Mf-MLAs was simply performed by a single photolithography step using a multi-sized hole lithography mask and the chemical wet etching step sequentially. Unlike conventional approaches to Mf-MLA, all the lens surface of newly fabricated Mf-MLAs successfully maintains the hemispherical shape by using isotropic etched properties of the quartz substrate, thus achieving a high NA of the Mf-MLA. Individual lens parameters are also elaborately handled by the wet etch time and the diameter of the hole. In addition, the fabricated Mf-MLAs clearly present the extended DoF ranges through the captured image at the multifocal plane. Therefore, the Mf-MLAs, as an essential micro-optics, are expected to pave the way for the advanced imaging systems such as 3D telemedicine endoscopes, holographic display, and the unmanned vehicles.

## Figures and Tables

**Table 1:** Surface roughness of Mf-MLAs with each type of microlens (cutoff: 8 μm).

Hole Size	2 μm	6 μm	10 μm
Roughness (R_a_)	0.044 μm	0.036 μm	0.034 μm
